# Development of Cas13a-based assays for *Neisseria gonorrhoeae* detection and gyrase A determination

**DOI:** 10.1128/msphere.00416-23

**Published:** 2023-09-21

**Authors:** Lao-Tzu Allan-Blitz, Palak Shah, Gordon Adams, John A. Branda, Jeffrey D. Klausner, Robert Goldstein, Pardis C. Sabeti, Jacob E. Lemieux

**Affiliations:** 1 Division of Global Health Equity, Department of Medicine, Brigham and Women’s Hospital, Boston, Massachusetts, USA; 2 Broad Institute of Massachusetts Institute of Technology and Harvard, Boston, Massachusetts, USA; 3 Division of Infectious Diseases, Department of Medicine, Massachusetts General Hospital, Boston, Massachusetts, USA; 4 Department of Pathology, Massachusetts General Hospital, Boston, Massachusetts, USA; 5 Department of Population and Public Health Sciences, Keck School of Medicine, University of Southern California, Los Angeles, California, USA; Antimicrobial Development Specialists, LLC, Nyack, New York, USA

**Keywords:** *Neisseria gonorrhoeae*, antimicrobial resistance, diagnostics, CRISPR

## Abstract

*Neisseria gonorrhoeae* is one of the most common bacterial sexually transmitted infections. The emergence of antimicrobial-resistant *N. gonorrhoeae* is an urgent public health threat. Currently, the diagnosis of *N. gonorrhoeae* infection requires expensive laboratory infrastructure, while antimicrobial susceptibility determination requires bacterial culture, both of which are infeasible in low-resource areas where the prevalence of infection is highest. Recent advances in molecular diagnostics, such as specific high-sensitivity enzymatic reporter unlocking (SHERLOCK) using CRISPR-Cas13a and isothermal amplification, have the potential to provide low-cost detection of pathogen and antimicrobial resistance. We designed and optimized RNA guides and primer sets for SHERLOCK assays capable of detecting *N. gonorrhoeae* via the *por*A gene and of predicting ciprofloxacin susceptibility via a single mutation in the gyrase A (*gyr*A) gene. We evaluated their performance using both synthetic DNA and purified *N. gonorrhoeae* isolates. For *por*A*,* we created both a fluorescence-based assay and lateral flow assay using a biotinylated fluorescein reporter. Both methods demonstrated sensitive detection of 14 *N*. *gonorrhoeae* isolates and no cross-reactivity with 3 non-gonococcal *Neisseria* isolates. For *gyr*A, we created a fluorescence-based assay that correctly distinguished between 20 purified *N. gonorrhoeae* isolates with phenotypic ciprofloxacin resistance and 3 with phenotypic susceptibility. We confirmed the *gyr*A genotype predictions from the fluorescence-based assay with DNA sequencing, which showed 100% concordance for the isolates studied. We report the development of Cas13a-based SHERLOCK assays that detect *N. gonorrhoeae* and differentiate ciprofloxacin-resistant isolates from ciprofloxacin-susceptible isolates.

**IMPORTANCE:**

*Neisseria gonorrhoeae,* the cause of gonorrhea, disproportionately affects resource-limited settings. Such areas, however, lack the technical capabilities for diagnosing the infection. The consequences of poor or absent diagnostics include increased disease morbidity, which, for gonorrhea, includes an increased risk for HIV infection, infertility, and neonatal blindness, as well as an overuse of antibiotics that contributes to the emergence of antibiotic resistance. We used a novel CRISPR-based technology to develop a rapid test that does not require laboratory infrastructure for both diagnosing gonorrhea and predicting whether ciprofloxacin can be used in its treatment, a one-time oral pill. With further development, that diagnostic test may be of use in low-resource settings.

## INTRODUCTION

*Neisseria gonorrhoeae* is one of the most common bacterial sexually transmitted infections worldwide (1). There were an estimated 87 million cases reported in 2016 (1), with the highest prevalence among low-resource settings (2 – 4), which is likely to be an underestimate due to under-reporting. The consequences of inadequately treated infection can be serious, ranging from pelvic inflammatory disease (5), infertility (6), and neonatal blindness (7), to an increased risk for HIV infection (8 – 13).

Furthermore, antimicrobial resistance in *N. gonorrhoeae* is a global public health threat (14, 15). *N. gonorrhoeae* has developed resistance to nearly all antimicrobials used in its treatment (16). Because culture is not routinely performed and standard-of-care nucleic acid amplification testing via polymerase chain reaction (PCR) does not provide information on antibiotic susceptibility, all *N. gonorrhoeae* infections in the United States are treated with third-generation cephalosporins, further driving selective pressure toward the emergence of resistance (16, 17). Recent reports of resistance to third-generation cephalosporins (18 – 22) have raised concern for untreatable infection. In response, the U.S. Centers for Disease Control and Prevention has increased the recommended dose of ceftriaxone for treating gonorrhea (23). However, the treatment of *N. gonorrhoeae* infection with antibiotics no longer empirically recommended due to high levels of resistance has been made possible by rapid molecular assays detecting genotypic markers of resistance (16, 17, 24). Use of such assays might reduce the spread of cephalosporin resistance (25).

Neither PCR for pathogen detection nor bacterial culture for susceptibility determination is available in most low-resource settings, as PCR requires expensive laboratory infrastructure and culture can be laborious and time intensive for *N*. gonorrhoeae (26). Consequently, the treatment of *N. gonorrhoeae* infection is limited to syndromic management in low-resource settings, which is insensitive for case finding (27 – 29) and further drives the emergence of antimicrobial resistance (16, 17). In fact, limited data suggest that low-resource areas have some of the highest prevalence of antimicrobial-resistant *N. gonorrhoeae* infections (30 – 32). Thus, the World Health Organization’s action plan for combating the emergence of antimicrobial resistance calls for the development of rapid molecular assays for pathogen detection and predicting antimicrobial susceptibility (33). Previous work has indicated that the *por*A gene may be a useful target for *N. gonorrhoeae* detection (34) and that phenotypic resistance to ciprofloxacin is predicted by the presence of a single-nucleotide polymorphism at codon 91 of the gyrase A (*gyr*A) gene (35, 36). Such testing, however, still requires PCR capabilities, which are generally inaccessible in low-resource settings.

Specific high-sensitivity enzymatic reporter unlocking (SHERLOCK) technology utilizes Cas13a, a CRISPR enzyme paired with isothermal amplification via recombinase polymerase amplification (RPA) (37, 38), a low-cost, sensitive, and field-deployable diagnostic technology (39, 40). Cas13a-based detection works via complementary binding of programmable CRISPR guide RNA (gRNA) sequences to target sequences, which activates the inherent Cas13a-mediated collateral cleavage of an RNA reporter (37, 41). Such assays can be employed with standard fluorescence reports or adapted for paper-based lateral flow detection (42). Moreover, Cas13a has been shown to have reduced tolerance for activation with increasing mismatches between gRNA and the template, which can facilitate discriminating between strains containing point mutations. In this study, we aimed to develop SHERLOCK assays for *N. gonorrhoeae* detection and *gyr*A genotype determination. We explored fluorescence-based and lateral flow readouts for each assay and evaluated their performance using *N. gonorrhoeae* synthetic DNA and purified isolates. We aimed for this work to be a first step toward developing methods for *N. gonorrhoeae* detection and antimicrobial resistance determination accessible anywhere in the world.

## MATERIALS AND METHODS

### Reagents and materials

Detailed information on reagents used and stock concentrations can be found in Tables S1 and S2.

### Synthetic DNA preparation and DNA extraction from purified isolates

We tested assays using both synthetic *N. gonorrhoeae* DNA and purified *N. gonorrhoeae* isolates. We prepared synthetic DNA samples by serial dilution from commercially purchased (Integrated DNA Technologies, USA), double-stranded DNA (dsDNA) of the *gyr*A target region into nuclease-free water. We stored purified isolates in glycerol at −80°C prior to extraction. We extracted whole-genomic DNA from *N. gonorrhoeae* purified isolates using the DNeasy Blood and Tissue Kit (Qiagen, Germany). The starting volume for extraction was 400 µL, and extracted DNA was eluted into 100 µL of nuclease-free water. With each isolate, we were provided minimum inhibitory concentrations (MICs) in micrograms per milliliter for ciprofloxacin, obtained using standard methods, as well as the anatomic site of collection (Table 1). Additionally, we purchased non-gonococcal *Neisseria* isolates from American Type Culture Collection (ATCC), and the Massachusetts General Hospital Clinical Microbiology Laboratory cultured those isolates: *N. meningitidis* (ATCC 13077)*, N. perflava* (ATCC 14799), and *N. lactamica* (ATCC 23970). The performance of the *por*A assay was also assessed on those isolates.

**TABLE 1:** Characteristics of purified *N. gonorrhoeae* isolates

Year collected	Anatomic site	Ciprofloxacin MIC (µg/mL)^*a*^	Resistance interpretation	*Gyr*A genotype (PCR)	*Gyr*A concordance
2014	Pharynx	≤0.015	Susceptible	Wild type	Yes
2014	Pharynx	≤0.015	Susceptible	Wild type	Yes
2014	Pharynx	≤0.015	Susceptible	Wild type	Yes
2014	Urethra	8.000	Resistant	Mutant	Yes
2013	Urethra	8.000	Resistant	Mutant	Yes
2013	Urethra	8.000	Resistant	Mutant	Yes
2013	Urethra	>16.000	Resistant	Mutant	Yes
2014	Urethra	>16.000	Resistant	Mutant	Yes
2014	Urethra	8.000	Resistant	Mutant	Yes
2014	Urethra	>16.000	Resistant	Mutant	Yes
2014	Urethra	8.000	Resistant	Mutant	Yes
2014	Urethra	8.000	Resistant	Mutant	Yes
2011	Urethra	16.000	Resistant	Mutant	Yes
2011	Urethra	16.000	Resistant	Mutant	Yes
2012	Urethra	16.000	Resistant	Mutant	Yes
2011	Urethra	16.000	Resistant	Mutant	Yes
2011	Urethra	16.000	Resistant	Mutant	Yes
2014	Urethra	>16.000	Resistant	Mutant	Yes
2014	Urethra	1.000	Resistant	Mutant	Yes
2014	Urethra	1.000	Resistant	Mutant	Yes
2013	Urethra	1.000	Resistant	Mutant	Yes
2014	Urethra	16.000	Resistant	Mutant	Yes
2013	Urethra	16.000	Resistant	Mutant	Yes

^*a*^ Minimum inhibitory concentration.

We quantified the concentration of extracted *N. gonorrhoeae* DNA using quantitative polymerase chain reaction (qPCR). The forward and reverse primer sequences for the *N. gonorrhoeae gyr*A gene were 5′ GCGACGGCCTAAAGCCAGTG 3′ and 5′ GTCTGCCAGCATTTCATGTGAG 3′, respectively. Those primers were provided by a previous study (43). The qPCR mixtures contained 1× FastStart SYBR Green Master Mix (Sigma Aldrich, USA), 0.5 µM of each primer, and DNA template in a 1:9 template to master mix ratio. We adjusted the final qPCR volume to 10 µL with nuclease-free water and loaded in triplicate on a 384-well plate, which was run on a QuantStudio 6 (Applied Biosystems, USA) with the following cycle conditions: heat activation at 95°C for 3 minutes, 40 cycles of a denaturing step at 95°C for 15 seconds, an annealing step at 60°C for 1 minute, and an extension step at 72°C, followed by a final extension step at 68°C for 2 minutes. We collected amplification data during the second extension stage and analyzed those data using the standard curve module of the Applied Biosystems Analysis Software. We quantified isolates against a standard curve, which showed an average concentration of 1,000 copies per milliliter across isolates. Subsequently, we evaluated thermal DNA extraction by resuspending three purified isolates in 100 µL of nuclease-free water and heating the isolates to 95°C for 10 minutes in accordance with prior protocols (44).

### Guide RNA and primer design for *N. gonorrhoeae* detection

Cas13a gRNAs have two components: the fixed “handle” region to which the Cas13a protein binds and a 28-nucleotide “spacer” region complementary to the target. The nucleotide sequence of the spacer can be chosen by the user to confer the specificity of the assay. We selected the *por*A gene of *N. gonorrhoeae* for pathogen detection as has been used previously (34). We used an online software package ADAPT (Activity-Informed Design with All-Inclusive Patrolling of Targets; https://adapt.run) (45), which applies an algorithm for optimal Cas13a gRNAs design, and selected three gRNAs from the output of that software targeting different locations in the *por*A gene.

We designed forward and reverse RPA primers using National Center for Biotechnology Information Primer-Basic Local Alignment Search Tool (BLAST), which were synthesized by Integrated DNA Technologies (USA). We developed two primer sets per guide location (total of six primer sets), which were 27–35 nucleotides in length. The primer sets had melting temperatures between 58°C and 68°C and produced amplicons of 140–200 base pairs in length. We appended a T7 RNA polymerase promoter sequence (5′ GAAATTAATACGACTCACTATAGG 3′) to the 5′ end of the forward primers of each set to allow for T7 transcription.

### One-pot SHERLOCK assay

We performed SHERLOCK reactions using 45 nM C2c2 *Lwa*Cas13a (GenScript Biotech Corp, USA) resuspended in 1× storage buffer (SB: 50 mM Tris [pH 7.5], 600 mM KCl, 5% glycerol, and 2 mM dithiothreitol [DTT]) such that the resuspended protein was at 2.25 µM, 1 U/µL murine RNase inhibitor (NEB), 10 U/µL T7 RNA polymerase (Lucigen Corporation, USA), 136 nM RNaseAlert substrate v2 (ThermoFisher Scientific, USA), 1× SHINE Buffer {SHINE: 20 mM HEPES [4-(2-hydroxyethyl)-1-piperazineethanesulfonic acid] (pH 8.0), 60 mM KCl, and 5% polyethylene glycol (PEG)}, and 2 mM of each rNTP (NEB).

We rehydrated the TwistAmp Basic Kit lyophilized pellets (one pellet per 73.42-µL master mix volume) using the prepared master mix. We added 14 mM MgAOc (TwistDx, United Kingdom) after resuspension to activate the RPA pellets. We then subdivided the master mix for each guide-primer set pair being analyzed, to which we added 22.5 nM gRNA (Integrated DNA Technologies, USA) and 320 nM each of the RPA primers (Integrated DNA Technologies, USA). We prepared SHERLOCK reactions to 70 µL and loaded as 20-µL triplicates into a 384-well plate, with a ratio of 1:5 master mix to sample. We measured fluorescence by the BioTek Cytation 5 plate reader (BioTek, USA) over 3 hours at 37°C, with readings every 5 minutes (excitation, 485; emission, 528) for quantitative detection.

### Lateral flow detection

To convert to lateral flow readout, we modified the SHERLOCK master mix to exchange substrate v2 for a biotinylated fluorescein (FAM) reporter at a final concentration of 1 µM. We incubated samples at 37°C for 90 minutes per existing protocols to allow for optimal RPA amplification. Following incubation, we added 80-µL HybriDetect assay buffer (Milennia Biotec, Germany) to each sample in a 1:5 dilution along with a HybriDetect lateral flow strip (Milennia Biotec, Germany). We inspected strips and took images using a smartphone camera 3–5 minutes after the strips were added.

### Confirmatory DNA sequencing

We performed whole-genome sequencing on extracted DNA samples following the Illumina DNA Prep manufacturer protocol (Illumina, USA). We constructed and pooled libraries using the Illumina DNA Prep Kit. We measured library concentrations on a Qubit4 Fluorometer using the Qubit High Sensitivity 1× dsDNA kit (ThermoFisher Scientific, USA), while we measured the average library size on an Agilent TapeStation 4150 using the Agilent High Sensitivity D1000 ScreenTape kit (Agilent Technologies, USA). We conducted genomic sequencing on an Illumina MiniSeq instrument (Illumina, USA).

### Data analysis

We subtracted baseline fluorescence (at 0 minutes) from fluorescence values through reaction progression. We averaged the final 10 fluorescence values of each replicate to provide the reported fluorescence values. We compared mean differences in fluorescence using Student’s *t*-test, with significance defined as *P* < 0.05. We interpreted lateral flow readouts by visual inspection. We generated all figures in PRISM Software version 9.5.1 (GraphPad, USA).

## RESULTS

### *N. gonorrhoeae* detection via a Cas13a-based *por*A assay

**Fig 1:**
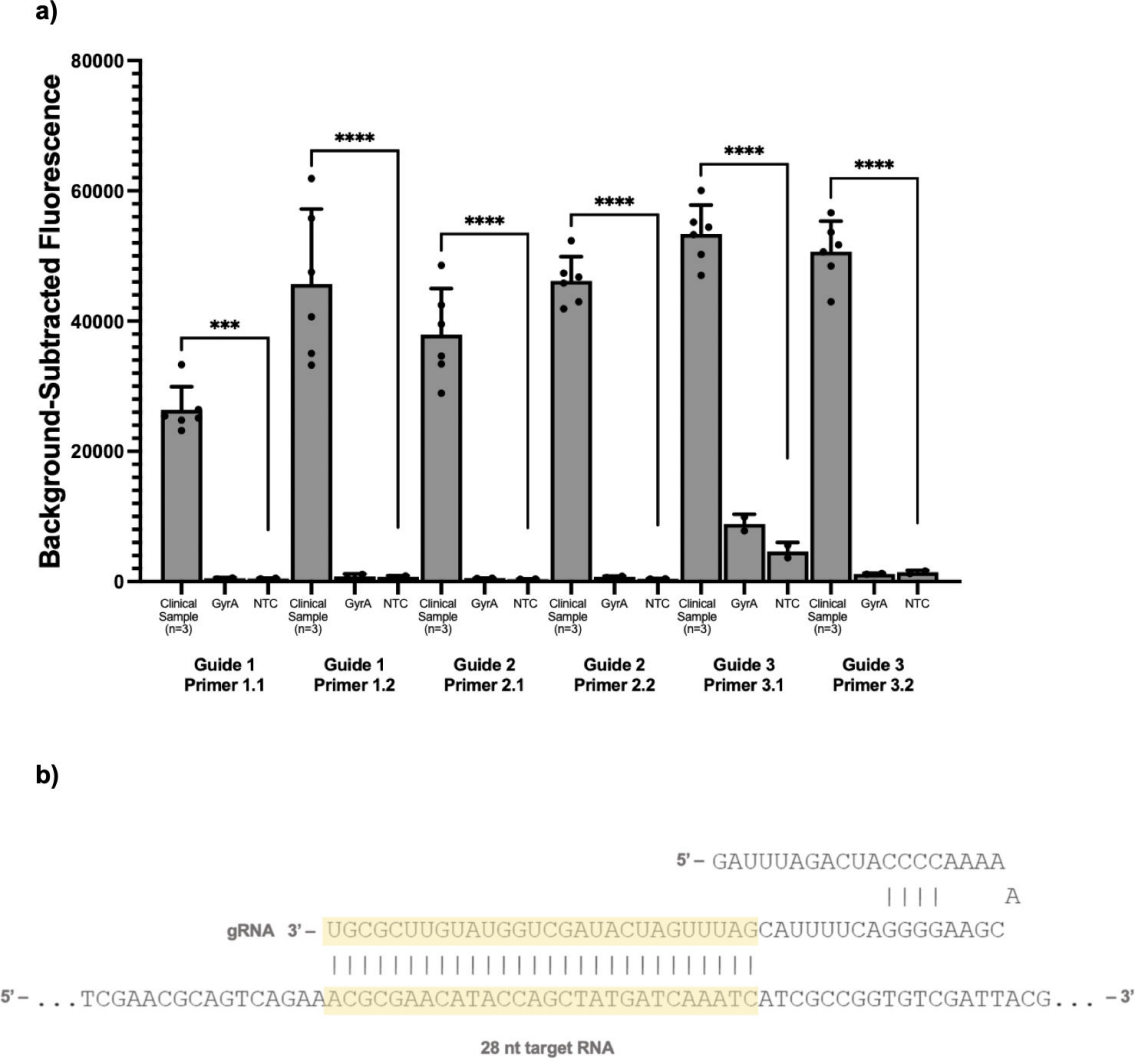
Guide and primer selection for a Cas13a-based assay for detecting *N. gonorrhoeae*. (**a**) Performance of three guides targeting different regions of the *por*A gene tested on three *N*. *gonorrhoeae* purified isolates as well as synthetic *gyr*A template as a control and a negative template control (NTC). (**b**) The selected *por*A guide sequence. *** indicates statistically significant differences in florescence at the *P* < 0.05 level.

To create an assay for *N. gonorrhoeae* detection, we first designed six *por*A primer-guide pairs and evaluated their performance, both in terms of high sensitivity and low cross-reactivity, using a fluorescence-based readout (Fig. 1). We performed initial testing on three purified *N. gonorrhoeae* isolates using both negative template controls as well as synthetic *gyr*A as a positive control. We selected guide 2 primer set 2 as it produced both a high fluorescent signal and excellent discrimination between synthetic *N. gonorrhoeae* purified isolates and the negative controls. We excluded guide 3 primer set 1 due to cross-reactivity with the *gyr*A control.

**Fig 2:**
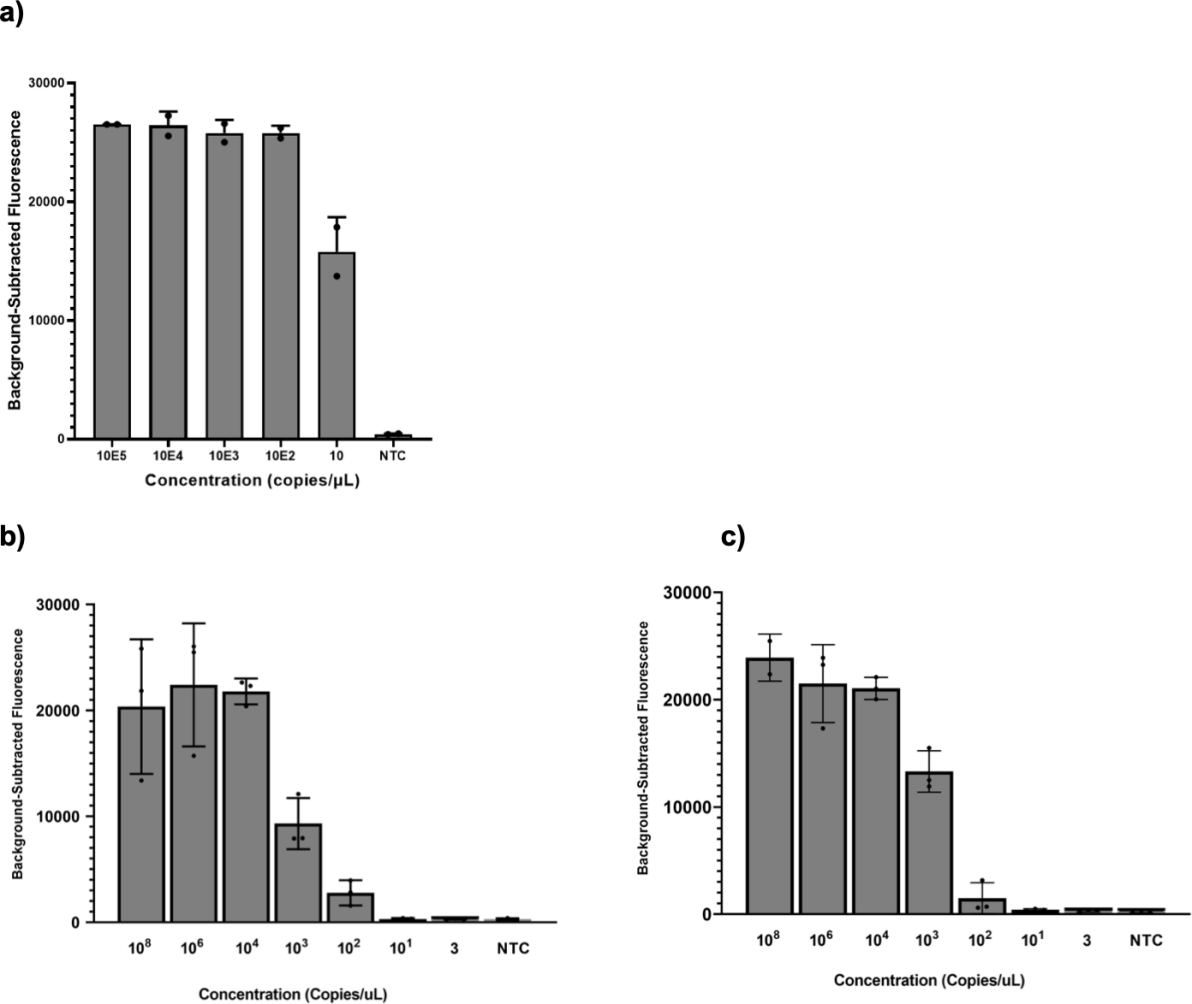
*In vitro* limit of detection of the Cas13a *N. gonorrhoeae* and *gyr*A genotypic assays. (**a**) The limit of detection of the *N. gonorrhoeae* Cas13a detection assay using the selected guide-primer set for the *por*A gene among purified *N. gonorrhoeae* isolates and a negative template control (NTC). (**b**) The limit of detection of the Cas13a-based assay using the wild-type guide against synthetic wild-type DNA target. (**c**) The limit of detection of Cas13a-based assay using the mutant guide against synthetic mutant DNA target. The serial dilutions of synthetic DNA were done in nuclease-free water.

Having selected our gRNA and primer set for *por*A detection, we evaluated the limit of detection (LoD) using serial dilutions in nuclease-free water as well as the detection of purified *N. gonorrhoeae* isolates using a fluorescence-based readout. The *por*A assay had an LoD of 10,000 copies per milliliter (Fig. 2a). We then tested the assay on 14 purified isolates and 3 non-gonococcal *Neisseria* isolates: *N. meningitidis, N. perflava,* and *N. lactamica*. The assay detected all 14 *N. gonorrhoeae* isolates, with peak fluorescence occurring after 20 minutes and did not detect any of the non-gonococcal *Neisseria* isolates.

**Fig 3:**
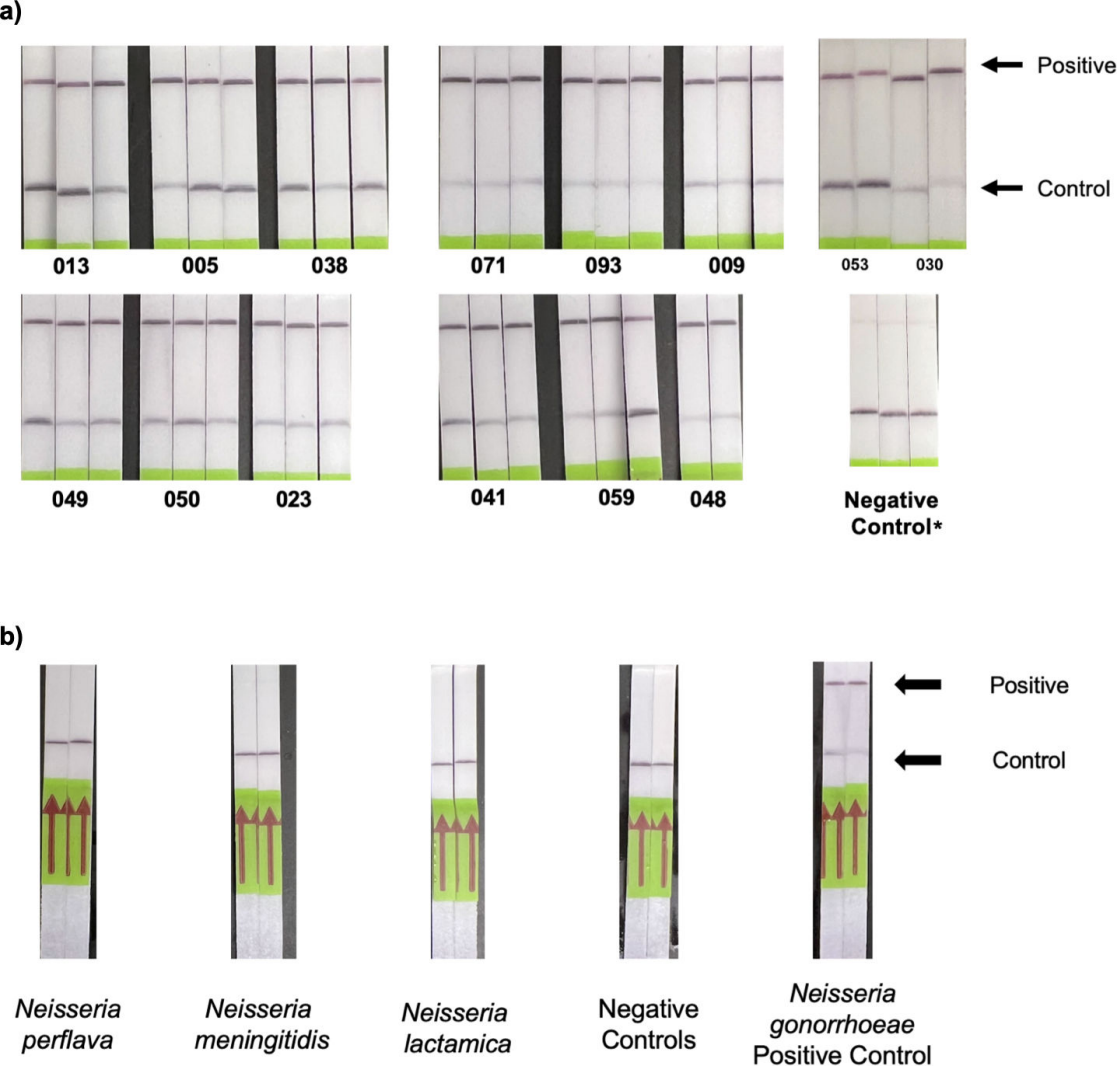
Performance of a Cas13a-based lateral flow assay for detecting *N. gonorrhoeae*. (**a**) The performance of the Cas13a-based lateral flow assay on 14 purified *N. gonorrhoeae* isolates tested in triplicate. (**b**) The discrimination of the lateral flow assay for *N. gonorrhoeae* isolates compared with non-gonococcal *Neisseria* isolates. * indicates the faint band at the test line in the negative control is expected per the manufacturer protocol.

We then assessed the *N. gonorrhoeae por*A detection assay using a lateral flow readout, substituting the standard fluorescence reporter with a biotinylated FAM reporter compatible with the test strips. Based on prior protocols, we allocated 90 minutes for the assay. Visual inspection of the test strips 3–5 minutes after specimen introduction revealed detection of all 14 purified isolates tested in triplicate (Fig. 3a) and excellent discrimination between *N. gonorrhoeae* and three non-gonococcal *Neisseria* isolates (Fig. 3b).

Having shown that we can develop a lateral flow-based *N. gonorrhoeae* detection assay, we explored the possibility of simplifying upstream DNA extraction to facilitate deployment in low-resource settings. To do so, we evaluated fluorescence *N. gonorrhoeae* detection on three purified isolates that underwent thermal DNA extraction. We quantified the DNA extracted using PCR and found DNA concentrations above 1,000,000 copies per milliliter. All three of those isolates were detected using the selected guide-primer set combination.

### *Gyr*A genotype determination via a Cas13a-based assay

To create an assay for predicting *N. gonorrhoeae* resistance to ciprofloxacin, we first designed two guide pairs (wild type and mutant) to target the point mutation in codon 91 of the *gyr*A gene and three flanking primer sets. We placed the mutation of interest three nucleotides distal to the Cas hairpin, previously shown to be the optimal position (46). We placed an additional synthetic mismatch in either the second position or the fourth position of the spacer region. We elected to design the guides manually instead of using ADAPT, given the precise mutation of interest was known. Placing the synthetic mutation at the second position produced the highest fluorescence and greatest discrimination between the wild-type and mutant synthetic DNA targets (Fig. S1). We tested three forward and reverse primer sets for use with that guide and selected the set that produced the highest fluorescence signal and greatest discrimination between the wild-type and mutant synthetic DNA targets (Fig. S2). We evaluated the *in vitro* LoD of the fluorescence-based *gyr*A assay via serial dilutions in nuclease-free water of synthetic wild-type and mutant DNA targets. The *gyr*A assay had an LoD of 1,000,000 copies per milliliter for both wild-type and mutant targets (Fig. 2b).

To further assess the performance of the *gyr*A assay, we analyzed 23 purified *N. gonorrhoeae* isolates with susceptibility to ciprofloxacin determined phenotypically by culture and genotypically by sequencing to detect mutation codon 91 of the *gyr*A gene. We used a standard MIC breakpoint of ≥1 µg/mL to define ciprofloxacin resistance (Table 1) (47). Of the 23 isolates, 20 with MICs between 1 and >16 µg/mL were deemed resistant, and three with MICs <0.015 µg/mL were deemed susceptible. Of the 20 *N. gonorrhoeae* isolates with MICs ≥1 µg/mL, 100% had mutant *gyr*A genotypes by DNA sequencing. Of the 3 *N. gonorrhoeae* isolates with MICs <0.015 µg/mL, 100% had no mutation at codon 91 of the *gyr*A gene by DNA sequencing. Figure S3 shows the phylogenetic tree of the 23 *N. gonorrhoeae* isolates, demonstrating that the phylogenetically diverse isolates on which the Cas13a-based assay was tested.

**Fig 4:**
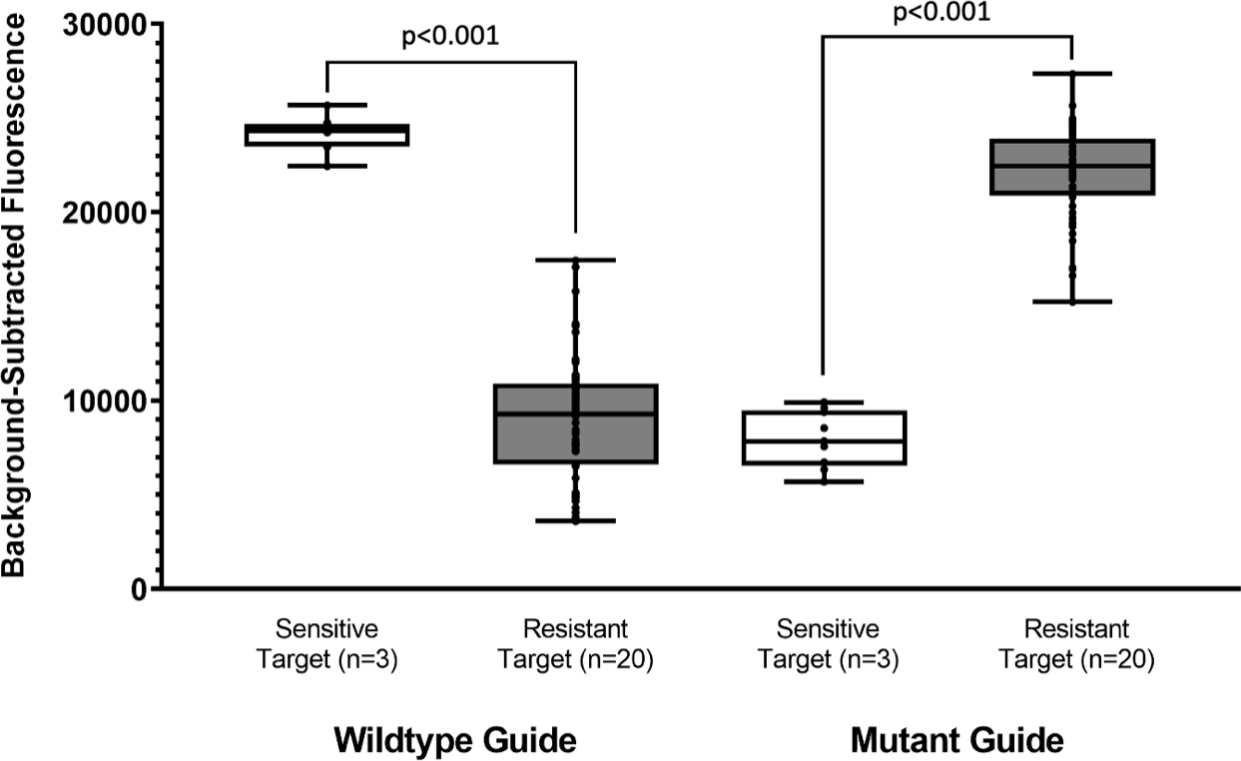
Cas13a-based gyrase A determination of purified *N. gonorrhoeae* specimens pooled discrimination of the Cas13a-based assay using fluorescence detection for determining the *gyr*A genotype of 23 purified *N. gonorrhoeae* isolates.

**Fig 5:**
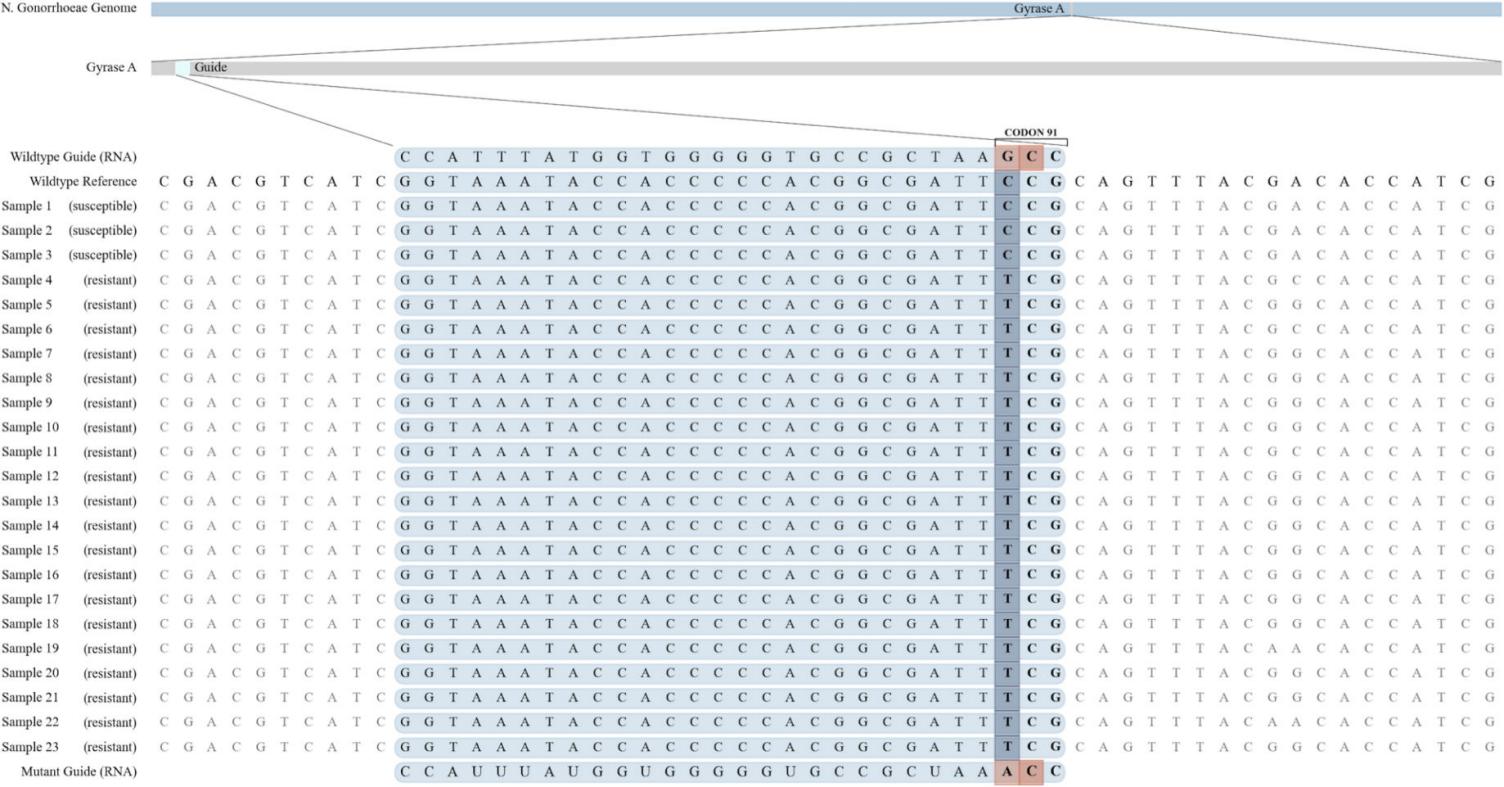
DNA sequence alignment of codon 91 of the *gyr*A gene from 23 purified *N. gonorrhoeae* isolates DNA sequence alignment of codon 91 of the *gyr*A gene in *N. gonorrhoeae* with the two CRISPR-Cas13a guide sequences.

We evaluated the discrimination of the selected wild-type and mutant Cas13a guides for codon 91 of the *gyr*A gene among all 23 isolates. All of the 20 ciprofloxacin-resistant *gyr*A mutant specimens were detected by the mutant Cas13a assay, while none of the three wild-type isolates were detected by the mutant Cas13a assay, showing a 100% agreement. Figure 4 shows the pooled performance among all specimens, while Fig. S4 shows the performance on each individual specimen. Figure 5 shows the DNA sequence alignment for all 23 isolates with the wild-type and mutant gRNAs.

**Fig 6:**
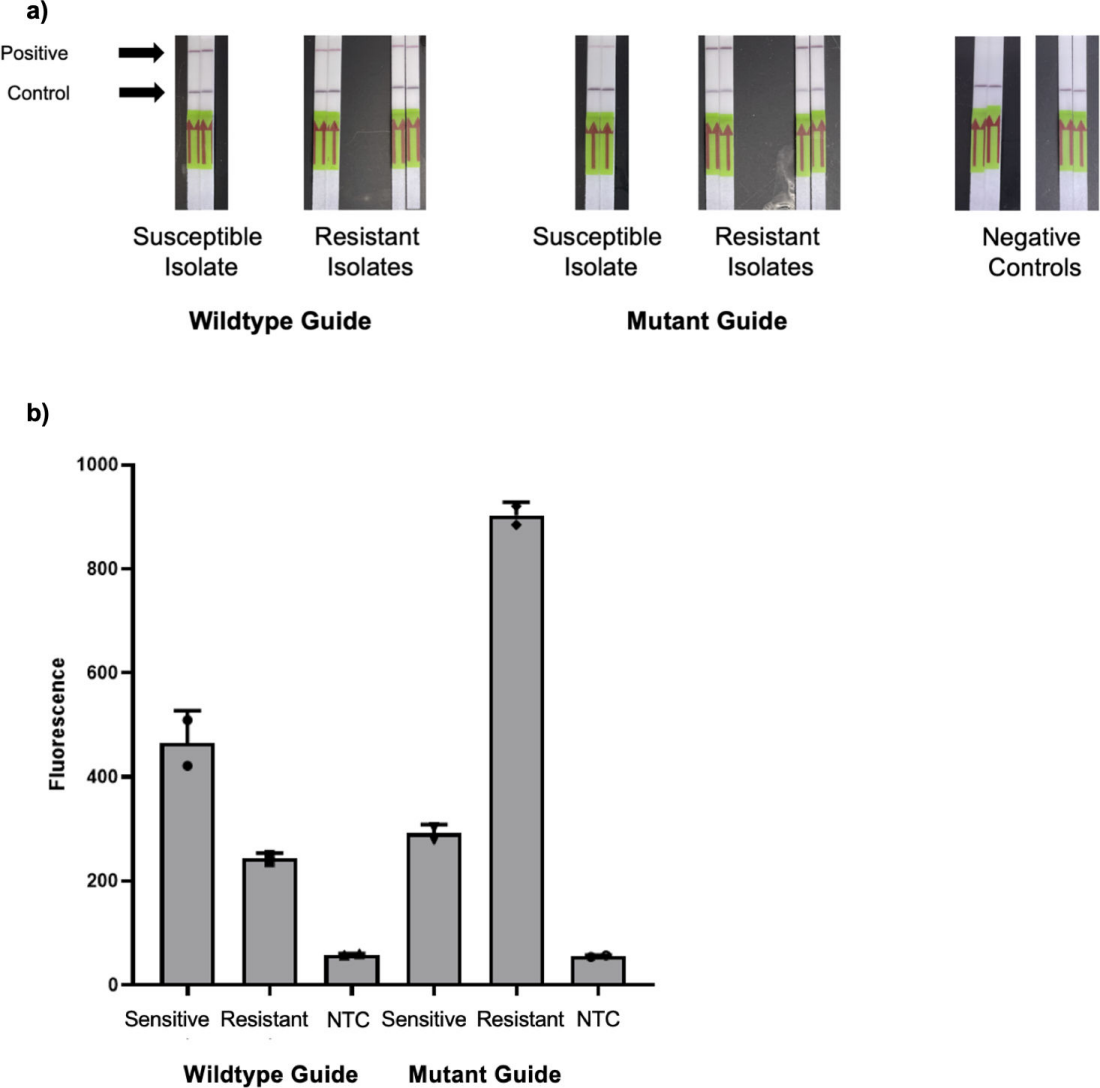
Performance of a Cas13a-based *gyr*A assay using lateral flow strips and a portable quantitative fluorometer on purified *N. gonorrhoeae* isolates. (**a**) Performance of a Cas13a-based lateral flow assay using both wild-type and mutant guides for determining *gyr*A genotype among 3 *N. gonorrhoeae* isolates. (**b**) The same Cas13a assay read on a Qubit 4 fluorometer. NTC, negative template control.

We next aimed to convert the *gyr*A resistance assay into a portable format suitable for use in resource-limited settings. We tested a lateral flow format, again substituting the standard fluorescence reporter with a biotinylated FAM reporter compatible with the test strips. Figure 6a shows the performance of the *gyr*A lateral flow on three purified isolates (one with known phenotypic and genotypic susceptibility to ciprofloxacin and two with known resistance). We tested each isolate in duplicate. The wild-type guide failed to discriminate visually between resistant and susceptible isolates. The mutant guide demonstrated promising discrimination; however, we detected a faint positive line in the susceptible isolate.

Given the technical limitations of our *gyr*A assay using a lateral flow readout, we evaluated the performance of the assay using a portable quantitative fluorescence detector. Such a detector, the Qubit 4 Fluorometer (ThermoFisher Scientific, USA), would permit low-cost detection in the absence of a plate reader (Cytation 5, BioTek, USA). We incubated our one-pot SHERLOCK reaction for 90 minutes at 37°C and then transferred the reaction to Qbit Assay tubes, diluted with nuclease-free water to 200 µL. We measured green fluorescence detection on the blue excitation setting (430–495 excitation filter; 510–580 emission filter). Figure 6b shows successful discrimination for both the wild-type and mutant isolates using that method.

## DISCUSSION

We report the development of a Cas13a-based lateral flow *N. gonorrhoeae* detection assay able to detect 100% of tested isolates, which did not amplify closely related *Neisseria* species. That assay offers the potential to introduce pathogen-specific diagnostics into low-resource settings that lack infrastructure for complex laboratory-based testing. More work is needed to establish the sensitivity and specificity of the assay in a clinical setting and to optimize its performance to meet World Health Organization standards for point-of-care tests (48). That includes the development of methods that could omit an extraction step and minimize time to detection. Our preliminary results indicate that thermal extraction is a promising strategy. While 90 minutes was allocated for the lateral flow incubation to standardize our findings with prior protocols, peak fluorescence was noted at 20 minutes, indicating that the assay could provide rapid results in the field.

We also report the development of a Cas13a-based fluorescence detection assay with excellent discrimination of wild-type and mutant *gyr*A genotype isolates for predicting ciprofloxacin resistance. That assay showed a 100% agreement with both phenotypically and genotypically determined resistance to ciprofloxacin. Given the urgent need to combat antimicrobial-resistant *N. gonorrhoeae* infections (14, 15) and the high burden of resistance in resource-limited settings (30 – 32), such an assay may permit resistance-guided therapy without expensive laboratory equipment. While promising, the lateral flow Cas13a *gyr*A assay was not able to discriminate between wild-type and mutant genotypes as definitively and will require further optimization. Iterative adaptations of guide sequences and position of the mutation of interest and of the synthetic mutation relative to the Cas hairpin may improve the specificity of the assay for the mutant *gyr*A genotype on the lateral flow platform. Additional optimization will also be required to reduce the time involved in running the assay.

As an alternative field-deployable method for determining ciprofloxacin resistance, we devised a method for portable fluorescence of *gyr*A genotypes that overcame the limitations of the lateral flow format for that assay. The portable fluorometer Cas13a *gyr*A assay showed excellent discrimination between sensitive and resistant genotypes and can be implemented in resource-limited settings much more easily than qPCR or the BioTek Cytation 5 plate reader. While more expensive than paper-based assays and electricity dependent, the fluorescence-based approach would still permit rapid and portable *gyr*A genotyping of *N. gonorrhoeae* specimens. With minor modifications, such as lyophilization of reagents and optimization of reaction conditions, we believe that some resource-constrained areas with basic laboratory infrastructure could consider assessing the feasibility of *N. gonorrhoeae* detection and *gyr*A genotyping using that assay format.

Our study had several important limitations. First, while we report on the *in vitro* performance of two newly described assays, our study evaluated the performance of those assays on a small number of isolates, thus limiting the precision of our findings. Moreover, the clinical utility remains to be determined and requires evaluation in a clinical setting. The processing required of those specimens will be of particular relevance for low-resource settings with limited laboratory infrastructure. However, while other rapid diagnostics for sexually transmitted infections are increasingly available (49), none has been sufficiently low cost, timely, and user friendly to be optimally suited for low-resource settings, and few have attempted to incorporate detection of molecular markers of resistance (50). Thus, our results may provide the groundwork for introducing point-of-care resistance-guided therapy into settings previously constrained to syndromic management.

### Conclusion

We developed a paper-based lateral flow Cas13a assay for detecting *N. gonorrhoeae*, which was able to detect *N. gonorrhoeae* purified isolates and discriminate between other *Neisseria* species. We also developed a fluorescence-based Cas13a assay for determining *gyr*A genotype, which demonstrated excellent discrimination for both phenotypic and genotypic ciprofloxacin resistance among purified isolates.

## SUPPLEMENTAL MATERIAL

Supplement msphere.00416-23-s0001.docx Tables S1 and S2; Figures S1–S4.

ASM does not own the copyrights to Supplemental Material that may be linked to, or accessed through, an article. The authors have granted ASM a non-exclusive, world-wide license to publish the Supplemental Material files. Please contact the corresponding author directly for reuse.
